# Effect of Berberine on promoting the excretion of cholesterol in high-fat diet-induced hyperlipidemic hamsters

**DOI:** 10.1186/s12967-015-0629-3

**Published:** 2015-08-27

**Authors:** Xiao-Yang Li, Zhen-Xiong Zhao, Min Huang, Ru Feng, Chi-Yu He, Chao Ma, Shi-Heng Luo, Jie Fu, Bao-Ying Wen, Long Ren, Jia-Wen Shou, Fang Guo, Yangchao Chen, Xin Gao, Yan Wang, Jian-Dong Jiang

**Affiliations:** State Key Laboratory of Bioactive Substance and Function of Natural Medicines, Institute of Materia Medica, Chinese Academy of Medical Sciences/Peking Union Medical College, Beijing, 100050 China; Beijing Analytical Application Center, Shimadzu (China) Co., Ltd., Beijing, 100020 China; Institute of Medicinal Biotechnology, Chinese Academy of Medical Sciences/Peking Union Medical College, Beijing, 100050 China; Department of Endocrinology and Metabolism, Zhongshan Hospital, Fudan University, Shanghai, 200032 China; School of Biomedical Sciences, Faculty of Medicine, The Chinese University of Hong Kong, Shatin, N.T. Hong Kong, China

**Keywords:** Berberine, Cholesterol, Hyperlipidemia, Mechanism, Excretion, Bile

## Abstract

**Background:**

Berberine (BBR), as a new medicine for hyperlipidemia, can reduce the blood lipids in patients. Mechanistic studies have shown that BBR activates the extracellular-signal regulated kinase pathway by stabilizing low-density-lipoprotein receptor mRNA. However, aside from inhibiting the intestinal absorption of cholesterol, the effects of BBR on other metabolic pathways of cholesterol have not been reported. This study aimed to investigate the action of BBR on the excretion of cholesterol in high-fat diet-induced hyperlipidemic hamsters.

**Methods:**

Golden hamsters were fed a high-fat diet (HFD) for 6 weeks to induce hyperlipidemia, followed by oral treatment with 50 and 100 mg/kg/day of BBR or 10 and 30 mg/kg/day of lovastatin for 10 days, respectively. The levels of total cholesterol (TC), triglyceride (TG), low-density lipoprotein cholesterol (LDL-C), transaminases, and total bile acid in the serum, liver, bile and feces were measured using an enzyme-linked immunosorbent assay. The cholesterol (as well as coprostanol) levels in the liver, bile and feces were determined by gas chromatography–mass spectrometry.

**Results:**

The HFD hamsters showed significantly hyperlipidemic characteristics compared with the normal hamsters. Treatment with BBR for 10 days reduced the serum TC, TG and LDL-C levels in HFD hamsters by 44–70, 34–51 and 47–71 %, respectively, and this effect was both dose- and time-dependent. Initially, a large amount of cholesterol accumulated in the hyperlipidemic hamster livers. After BBR treatment, reductions in the liver cholesterol were observed by day 3 and became significant by day 7 at both doses (*P* < 0.001). Meanwhile, bile cholesterol was elevated by day 3 and significantly increased at day 10 (*P* < 0.001). BBR promoted cholesterol excretion from the liver into the bile in hyperlipidemic hamsters but not in normal hamsters, and these results provide a link between the cholesterol-lowering effect of BBR with cholesterol excretion into the bile.

**Conclusions:**

We conclude that BBR significantly promoted the excretion of cholesterol from the liver to the bile in hyperlipidemic hamsters, which led to large decreases in the serum TC, TG and LDL-C levels. Additionally, compared with lovastatin, the BBR treatment produced no obvious side effects on the liver function.

## Background

Berberine (BBR) is a medicinal alkaloid that is isolated from *Coptis chinensis* and has been used orally for decades in China as a safe over-the-counter (OTC) drug to treat diarrhea [1]. In recent years, in addition to its antibacterial effect [2–5], this natural compound has been increasingly studied for its effects against various diseases, including hyperlipidemia [6], diabetes [7, 8], and nervous system [9] and cardiovascular diseases [10–12]. BBR has obvious effects on lowering the plasma total cholesterol (TC), triglyceride (TG) and low-density lipoprotein cholesterol (LDL-C) levels, and these effects may be better in some aspects than those achieved when using simvastatin and atorvastatin [13]. The mechanism of its cholesterol-lowering activity was found to be related to its effects on elevating the low-density lipoprotein receptor (LDLR) expression by stabilizing LDLR message ribonucleic acid (mRNA) and was completely distinct from the statin mechanism of inhibiting 3-hydroxy-3-methylglutaryl-coenzyme A (HMG-CoA) reductase [1]. In addition, the same groups conducted further studies, mainly in HepG2 cells, and observed similar effects of BBR [14, 15]. Since then, an increasing number of studies in numerous different rodent models, cells and patients with hyperlipidemia have been conducted. In addition to up-regulating the LDLR, BBR inhibited lipid synthesis in human hepatocytes via the activation of AMP-activated protein kinase (AMPK) [16]. BBR was also reported to be effective in inhibiting 3T3-L1 adipocyte accumulation and differentiation through the peroxisome proliferator-activated receptor (PPAR) γ pathway, and these results suggested that BBR worked as an inhibitor of PPAR γ and α on multiple molecular targets [17, 18]. The blood free fatty acid (FFA) level was decreased, and the activity of lipoprotein lipase was increased in hyperlipidemic and insulin-resistant rats after the administration of BBR [19]. A recent study showed that BBR decreased the blood cholesterol level in hypercholesterolemic rats by inhibiting intestinal absorption, followed by decreasing enterocyte cholesterol uptake and secretion [20]. Apart from LDLR-mediated LDL-C clearance in the liver, cholesterol homeostasis includes several other biological processes, such as cholesterol absorption, cholesterol biosynthesis, cholesterol excretion, bile acid biosynthesis and secretion. However, in addition to the intestinal absorption of cholesterol, the effects of BBR on these metabolic pathways should be shown. We know that cholesterol excretion from the liver to the bile is collectively controlled by various factors. Therefore, to further elucidate the cholesterol-lowering mechanism of BBR, we asked whether BBR could impact this important process.

Recent pharmacology studies have indicated that BBR possesses a good cholesterol-lowering activity and is a safer treatment than other hypolipidemic drugs [21]; therefore, BBR has become an effective hypolipidemic drug with potential benefits for the treatment of hyperlipidemia and other metabolic syndromes. Considering the lack of evident side effects reported from the clinical use of BBR [22] and the reported side effects of statins, including muscle pain, fatigue, and weakness, accompanied by an increase in creatine phosphokinase (CPK) and liver transaminase [23–25], it is meaningful to compare the lipid-lowering efficacies of BBR and lovastatin as well as their side effects on the liver in our study. Based on the effective doses in hamsters (50 and 100 mg/kg/day) [1], we performed this study with high-fat diet-induced hyperlipidemic hamsters by treating them with BBR and lovastatin for 10 days, respectively, to study the effect of BBR on the excretion of cholesterol and provide further support for elucidating its cholesterol-lowering mechanism.

## Methods

### Chemicals and reagents

Berberine chloride, lovastatin and 5α-cholestane were purchased from J&K Scientific Ltd (Beijing, China). Cholesterol and coprostanol were supplied by the National Institute for the Control of Pharmaceutical and Biological Products (Beijing, China) and Shanghai Huicheng Biotechnology Co., Ltd. (Shanghai, China), respectively. The purity of all standards listed above was more than 98 %. A package of kits for CHO, TG, LDL-C, alanine aminotransferase (ALT), aspartate aminotransferase (AST) and γ-glutamyltransferase (GGT) were purchased from BioSino Bio-Technology & Science Inc. (Beijing, China). The total bile acid (TBA) assay kit was obtained from the Nanjing Jiancheng Bioengineering Institute (Nanjing, China). High-performance liquid chromatography-grade *n*-hexane, ethyl acetate and methanol were obtained from J&K Scientific Ltd (Beijing, China). The distilled water was Wahaha purified water (Hangzhou, China).

### Equipment

A Shimadzu gas chromatograph mass spectrometer (GC–MS, Shimadzu Cooperation, Kyoto, Japan), EnSpire multimode microplate reader (PerkinElmer, Waltham, MA, USA), THZ-100-type incubation shaker (Shanghai, China), TG18G-II desktop universal high-speed centrifuge (Hunan Kaida Scientific Instruments Co., Ltd, Hunan, China), XW-80A miniature vortex mixers (Jintan Super Blue Instrument Manufacturing Co., Jintan, Ltd, China) and a MD200-2 nitrogen sweep blowing instrument (Hangzhou Dian Sheng Instrument Co., Ltd, Hangzhou, China) were used.

### Animals and study design

Eight-week-old male Syrian Golden Hamsters weighing 110–140 g (Vital River Laboratory Animal Technology Co., Ltd., Beijing, China) were housed in a controlled environment (21 ± 2 °C, 12-h light/dark cycle) at six per cage with free access to food and water during the acclimatization and study periods. The animal usage and experimental protocols were approved by the Laboratories’ Institutional Animal Care and Use Committee of the Chinese Academy of Medical Sciences and Peking Union Medical College. The research was conducted in accordance with all guidelines and ethics of the Chinese Council on Animal Care.

One hundred and twenty-four hamsters were acclimatized for 7 days in their cages before the start of the study. All animals were then randomly separated into two groups according to their weights: the normal control group (thirty-four animals) and the hyperlipidemia model group (ninety animals). The animals that served as the normal control group were fed the standard diet ad libitum, whereas the hyperlipidemia model hamsters were fed a high-fat diet (HFD) daily for a period of 6 weeks to induce hyperlipidemia. The high-fat diet (Beijing HFK Bioscience Co., Ltd, China) was composed of 83.0 % standard diet, 15.0 % fat, 1.0 % cholesterol and 1.0 % sodium cholate [26].

At the end of 6 weeks, all animals were fasted for 12 h. Blood was collected, and the serum was obtained to measure the serum levels of TC, TG and LDL-C. The animals fed with a high fat diet showed significantly higher levels of TC, TG and LDL-C than the animals in the normal control group. According to their serum TC levels, the hamsters that were fed the normal diet (ND) were randomly allocated into two groups: 8 animals served as the ND control group, and 26 animals were treated with BBR (100 mg/kg/day). The hamsters that were fed the HFD were then randomly allocated into five groups: 8 animals served as the HFD control group, 26 animals were treated with BBR (50 mg/kg/day), 26 animals were treated with BBR (100 mg/kg/day), 15 animals were treated with lovastatin (10 mg/kg/day) and 15 animals were treated with lovastatin (30 mg/kg/day) [27].

All animals were orally administered the placebo, BBR or lovastatin, which was dissolved by saline into liquid suspension, in the morning for 10 days. After the animals were fasted for 12 h, the blood samples were obtained from posterior orbital venous plexus at 0, 3, 5, 7, and 10 days (n = 6 for each time point) by immediate centrifugation at 5,000 r/min for 5 min to obtain the serum samples. Feces were collected at the same time points. The hamsters were anesthetized for the collection of bile and liver samples. All samples were stored at −20 °C for the subsequent analyses.

### Serum lipid, ALT, AST and GGT levels

The concentration of TC, TG, LDL-C, ALT, AST and ALT in serum was determined at the end of the treatment using enzymatic kits.

### Cholesterol in bile, liver and feces

The liver samples obtained from the animals were washed with saline and dried. After weighing, they were homogenized with 2 volumes [v (mL)/w (g)] of saline. The bile and liver homogenates (50 μL) were extracted with 300 μL of ethyl acetate after the addition of 10 μL of an internal standard solution (100 μg/mL of 5α-cholestane in methanol) and 10 μL of methanol, respectively. To prepare the cholesterol standard curve, the bile and liver homogenates (infinitely diluted with saline, 50 μL) were extracted with 300 μL of ethyl acetate after the addition of 10 μL of an internal standard and 10 μL of a cholesterol standard working solution (prepared at a serial concentrations of 0.01, 0.1, 0.2, 1, 2, 10, 20, 100, and 200 μg/mL by dissolving in methanol), respectively. The samples were mixed using a vortex mixer for 20 min, followed by centrifuging at 5,000×*g* for 5 min. The supernatant was dried under N_2_ flow at 40 °C [28], and the residue was reconstituted with 200 μL of *n*-hexane and filtered through a 0.22-μm micropore membrane. An aliquot of 1 μL was injected into the GC–MS system.

The feces samples were triturated before weighing and dissolved with 10 volumes [v (mL)/w (g)] of methanol, followed by ultrasonic extraction for 30 min and centrifugation at 10,000 r/min for 10 min. The supernatant from the feces (200 μL) was extracted with 1 mL of ethyl acetate after the addition of 10 μL of an internal standard (100 μg/mL of 5α-cholestane), 10 μL of methanol and 500 μL of distilled water. To prepare the standard curve of cholesterol and coprostanol, the supernatants from the feces (infinitely diluted with methanol, 200 μL) were extracted with 1 mL of ethyl acetate after the addition of 10 μL of an internal standard and 10 μL of a cholesterol and coprostanol standard working solution (prepared at a serial concentrations of 0.5, 2.5, 5, 25, 50, 125, 250, and 500 μg/mL by dissolving in methanol), respectively. The samples were mixed using a vortex mixer for 20 min and centrifuged at 5,000×*g* for 5 min. The supernatant was dried under N_2_ flow at 40 °C, and the residue was reconstituted with 200 μL of *n*-hexane and filtered through a 0.22-μm micropore membrane. An aliquot of 1 μL was injected into the GC–MS system.

A GC–MS method was used to determine the cholesterol (as well as coprostanol) levels in bile, liver and feces. A capillary column (AT-1701, 30 m × 0.25 mm, and 0.25 μm phase thickness) was used, and high purity helium was used as a carrier gas at a constant flow rate of 1 mL/min. The temperature program started at 60 °C, was increased to 270 °C after 2 min at a rate of 20 °C/min, and was maintained at that temperature for 12.5 min. The sample was injected in a split-less mode. The injector temperature and transfer linear temperatures were set to 260 and 265 °C, respectively. The mass spectrometer was operated in the electron impact ionization mode with 70 eV, and the ion source temperature was set to 230 °C.

### TBA in bile, liver and feces

The concentration of TBA in bile, liver and feces was determined at the end of the treatment using enzymatic kits.

### Statistical analysis

The statistical analysis was performed using Graph Pad Prism 5 software (Graph Pad Software, San Diego, CA, USA). The data were analyzed by one-way analysis of variance (ANOVA) followed by Dunnett’s post hoc test or two-way ANOVA plus Bonferroni’s post-test, as appropriate. The results are presented as the mean ± standard deviation (S.D.). *P*-values equivalent to a significance level of 0.05 were considered statistically significant.

## Results and discussion

### Serum lipid levels

**Fig. 1:**
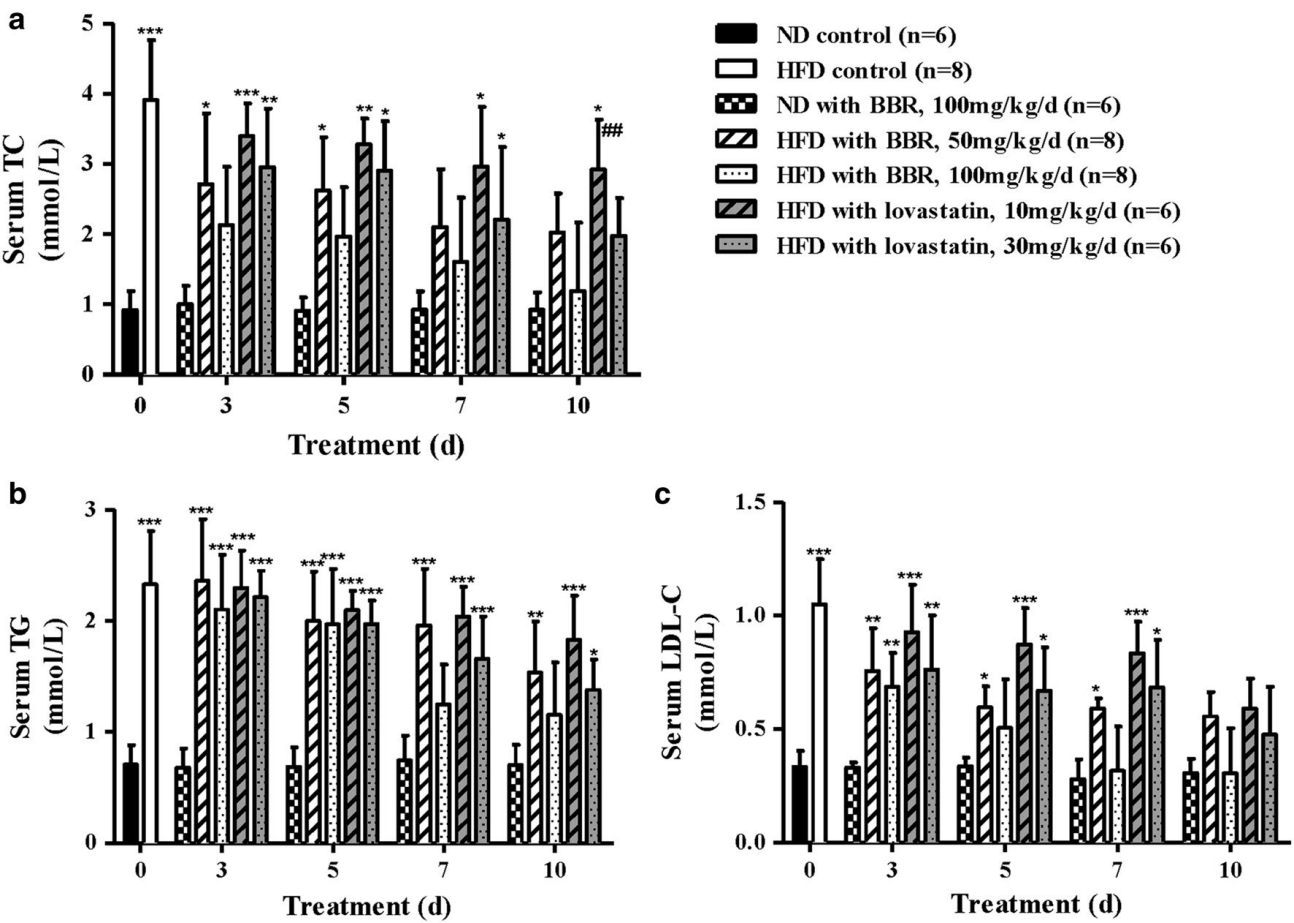
Serum lipid levels at each time point. (**a**) BBR and lovastatin significantly reduced the serum TC levels in the hyperlipidemic hamsters. The serum TC levels in the lovastatin-treated HFD hamsters (10 mg/kg/day) were significantly different from those of the BBR-treated HFD hamsters (100 mg/kg/day) at day 10. (**b**) BBR and lovastatin significantly reduced the serum TG levels in the hyperlipidemic hamsters. (**c**) BBR and lovastatin significantly reduced the serum LDL-C levels in the hyperlipidemic hamsters. The data are shown as the mean ± SD. *P < 0.05, **P < 0.01 and ***P < 0.001 vs the ND controls; ^##^ *P* < 0.01 vs the BBR-treated HFD hamsters (100 mg/kg/day).

At the end of 6 weeks, the serum TC, TG and LDL-C levels were increased by 3.86-, 3.32- and 2.94-fold (*P* < 0.001), respectively, in the HFD group compared with the normal control group (Table 1). The oral administration of BBR and lovastatin for 10 days resulted in dose- and time-dependent decreases in the serum TC, TG and LDL-C levels in the hyperlipidemic hamsters (Fig. 1a–c). After the 10-day treatment, 50 mg/kg/day BBR reduced the TC, TG and LDL-C levels in the hyperlipidemic hamsters by 44 % (*P* < 0.001), 34 % (*P* < 0.05), and 47 % (*P* < 0.001), respectively, and 100 mg/kg/day BBR reduced the TC, TG and LDL-C levels by 70 % (*P* < 0.001), 51 % (*P* < 0.001), and 71 % (*P* < 0.001), respectively, compared with the animals in the HFD control group. In contrast, 10 mg/kg/day lovastatin decreased the TC, TG and LDL-C levels by 25 % (*P* < 0.01), 21 %, and 44 % (*P* < 0.001), respectively, and 30 mg/kg/day lovastatin decreased the TC, TG and LDL-C levels by 49 % (*P* < 0.001), 41 % (*P* < 0.01), and 55 % (*P* < 0.001), respectively. At day 10, the serum TC levels in the lovastatin-treated HFD hamsters (10 mg/kg/day) were significantly different from those of the BBR-treated HFD (100 mg/kg/day) (*P* < 0.01). However, there was no significant difference among the TC, TG and LDL-C serum levels of animals in the BBR-treated ND group at each time point. The results described above show that BBR has an effective role in lowing the serum TC, TG and LDL-C levels and that BBR may be superior to lovastatin in lowering the serum TC levels. Additionally, BBR has no effect on the serum TC, TG and LDL-C levels in normal hamsters.

**Table 1:** Changes in the serum TC, TG and LDL-C levels in HFD hamsters after 6 weeks

	TC (mmol/L)	TG (mmol/L)	LDL-C (mmol/L)
Normal control	0.87	0.72	0.35
HFD	3.36***	2.39***	1.03***
Fold change	3.86	3.32	2.94

***p < 0.001 compared with the normal control group; Normal control animals, n = 20; HFD animals, n = 20. The statistical analysis was performed using a two-tailed Student’s *t* test.

### Serum ALT, AST and GGT levels

**Fig. 2:**
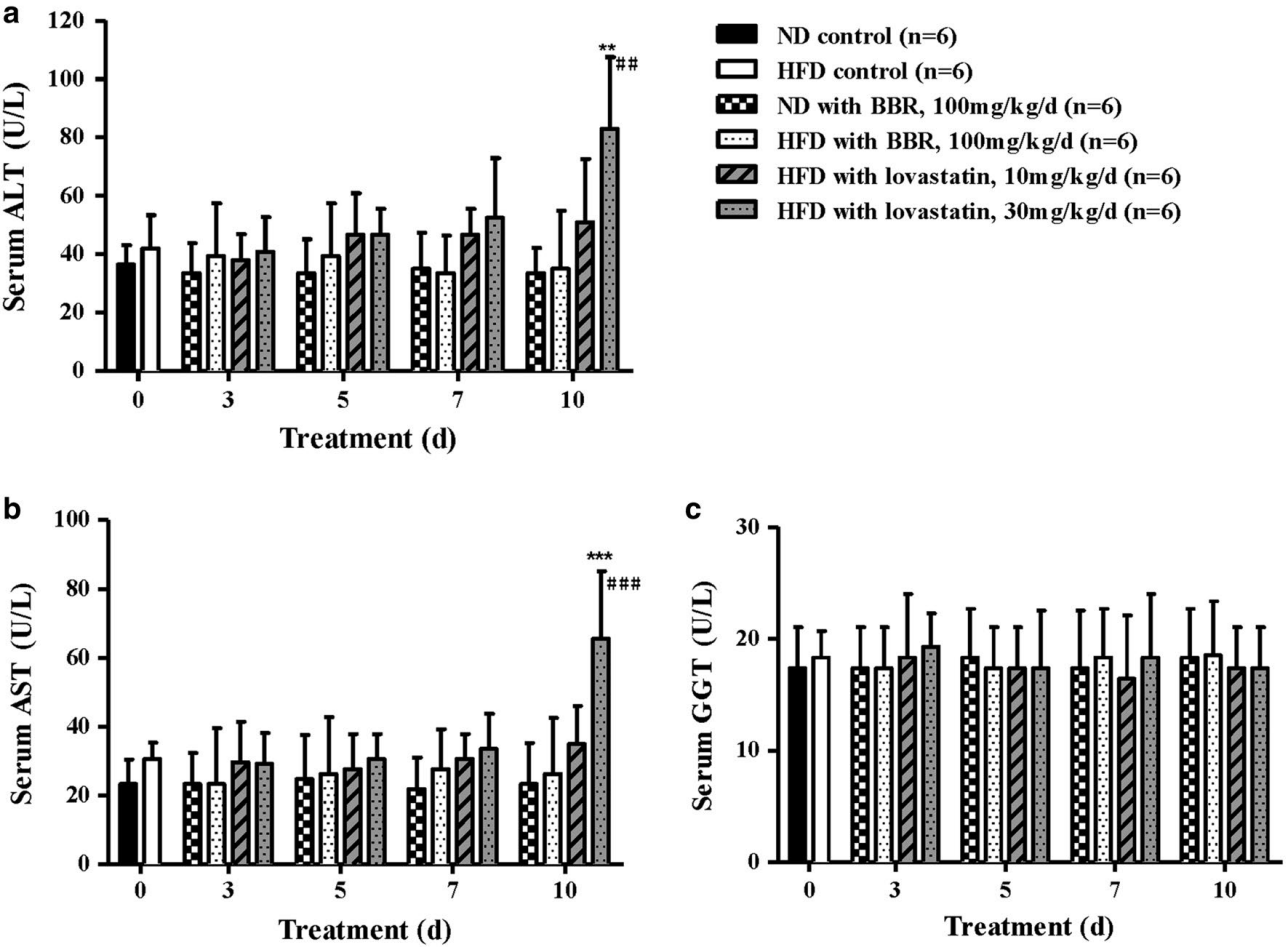
Effects of BBR and lovastatin on the liver function of hyperlipidemic hamsters. (**a**) Lovastatin (30 mg/kg/day) increased the serum ALT level by 2.0-fold at day 10 compared with the HFD control group at day 0, whereas the BBR (100 mg/kg/day) group showed no significant differences over the entire period. (**b**) Lovastatin (30 mg/kg/day) elevated the serum AST level by 2.1-fold at day 10 compared with the HFD control group at day 0, whereas the BBR (100 mg/kg/day) group showed no significant differences over the entire period. (**c**) There were no significant differences in the serum GGT levels in any of the groups. The data are shown as the mean ± SD. **P < 0.01 and ***P < 0.001 vs the HFD controls; ^##^ *P* < 0.01 and ^###^ *P* < 0.001 vs the BBR-treated HFD hamsters (100 mg/kg/day).

Measurements of the serum ALT, AST and GGT levels were performed to evaluate the effect of BBR and lovastatin on liver function. Significantly elevated serum ALT and AST levels were observed in the lovastatin-treated HFD hamsters (30 mg/kg/day) at day 10 compared with the HFD control group at day 0 (Fig. 2a, b), which were increased by 2.0-fold (*P* < 0.01) and 2.1-fold (*P* < 0.001), respectively, whereas the BBR-treated HFD group (100 mg/kg/day) showed no significant differences throughout the measurement period. The serum ALT and AST levels of the lovastatin-treated HFD group (30 mg/kg/day) were significantly different from those of the BBR-treated HFD group (100 mg/kg/day) at day 10 (*P* < 0.01 and *P* < 0.001, respectively). Thus, 30 mg/kg/day lovastatin treatment resulted in serum ALT and AST levels in the hyperlipidemic hamsters deviating from the normal range, which indicated that it caused a certain degree of liver damage. Nevertheless, our data show that no observable damage was caused by BBR in both the ND and HFD hamsters. BBR treatment even improved liver function by reducing the levels of ALT and ALT [1]. There was no significant difference among the groups in the serum GGT levels (Fig. 2c).

### Reduced cholesterol in the liver and increased cholesterol in bile

**Fig. 3:**
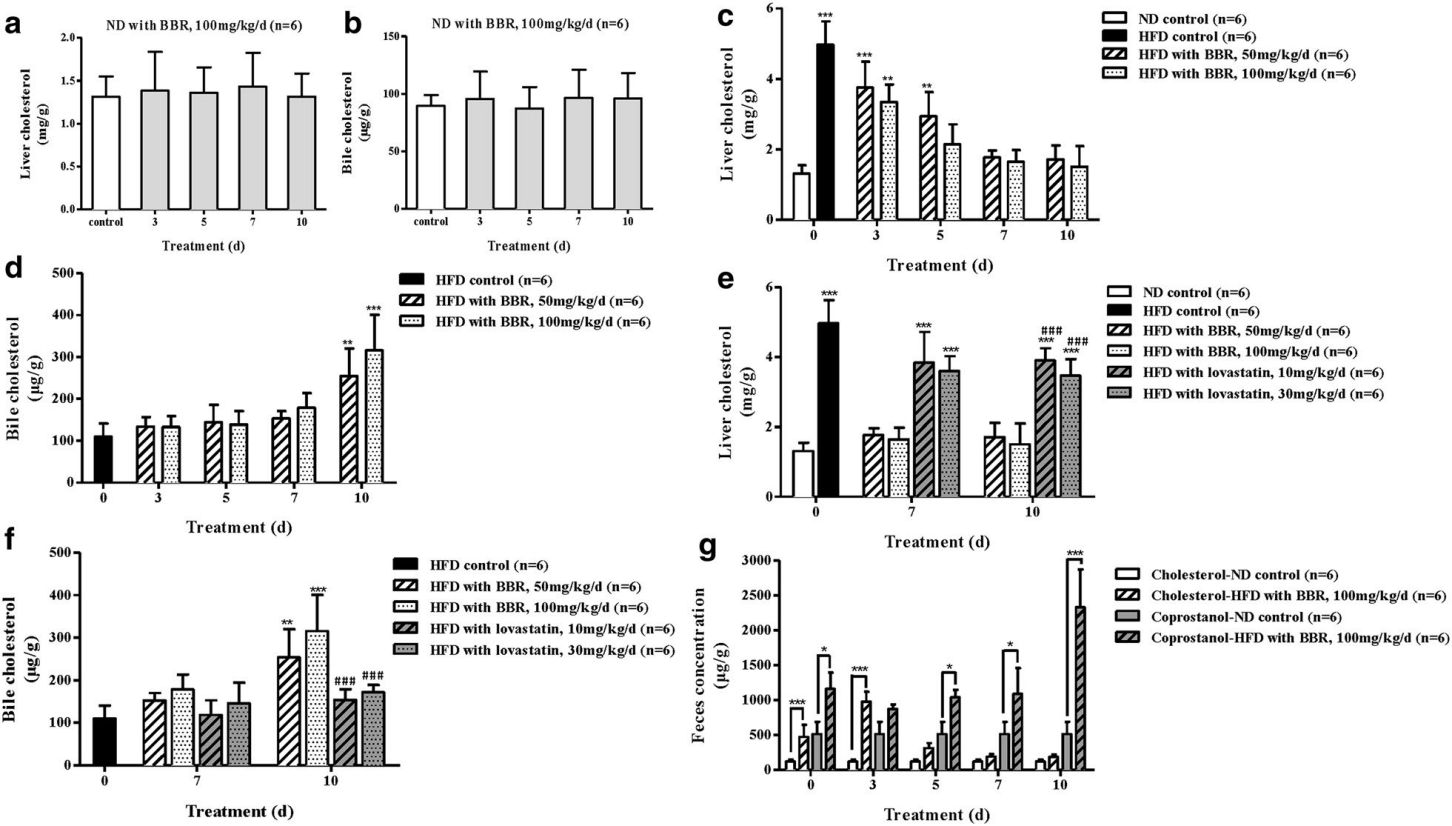
Reduced cholesterol in the liver and increased cholesterol in the bile after the BBR treatment. (**a**) The liver cholesterol levels in the BBR-treated ND hamsters. (**b**) The bile cholesterol levels in the BBR-treated ND hamsters. (**c**) Reductions in the liver cholesterol levels of the HFD hamsters were observed by day 3 and became significant by day 7 at both doses. (**d**) BBR resulted in significantly higher cholesterol levels in both BBR-treated HFD groups at day 10 than the HFD control group. (**e**) Lovastatin did not significantly decrease the cholesterol levels in the HFD hamster livers. (**f**) Lovastatin did not significantly increase the cholesterol levels in the HFD hamster bile. (**g**) In the feces, BBR decreased the levels of cholesterol, but significantly increased the levels of coprostanol from day 3 to day 10. The data are shown as the mean ± SD. *P < 0.05, **P < 0.01 and ***P < 0.001 vs the ND controls (**c**, **e** and **g**) or the HFD controls (**d**, **f**); ^###^ *P* < 0.001 vs the BBR-treated HFD hamsters (100 mg/kg/day).

This is the first study examining the cholesterol in bile in hyperlipidemic animals treated with BBR. To investigate the effect of BBR on cholesterol excretion in our study, both normal and hyperlipidemic hamsters were treated with BBR, and we determined the changes of cholesterol in the liver, bile and feces at each time point based on the results of the serum lipid levels. Similar to the serum lipid levels in normal hamsters, the cholesterol in the liver and bile in the BBR-treated ND group showed no significant differences throughout the measurement period (Fig. 3a, b), which indicated that BBR did not affect the cholesterol levels in the normal hamsters.

In the hyperlipidemic hamsters, the liver cholesterol level was significantly increased compared to the ND control hamsters (Fig. 3c). A large amount of cholesterol may be accumulated in the liver due to the high-fat diet, according to a previous study that used H&E staining of the liver tissue and showed obvious fat droplet accumulation in livers of HFD-fed hamsters [26]. After the BBR treatment, reductions in liver cholesterol in HFD hamsters were observed by day 3 and became significant by day 7 at both doses (*P* < 0.001). The bile cholesterol was elevated by day 3, and both BBR-treated HFD groups showed significantly higher bile cholesterol levels at day 10 compared with the HFD control group (*P* < 0.01 and *P* < 0.001, respectively, Fig. 3d). The BBR effect was dose-dependent in both the levels of liver and bile cholesterol. Moreover, the level of liver cholesterol in the HFD group at day 10 showed no significant differences compared with the ND control group at day 0 (Fig. 3c). We conclude that BBR has a remarkable effect on promoting cholesterol excretion from the liver into the bile in hyperlipidemic hamsters, and these results provide a link between the cholesterol-lowering effect of BBR with its activity in promoting cholesterol excretion into the bile. Additionally, the liver and bile cholesterol levels of both lovastatin-treated HFD hamsters were significantly different than the 100 mg/kg/day BBR-treated HFD hamsters at day 10 (*P* < 0.001). These results showed that lovastatin did not significantly decrease the liver cholesterol level or increase the bile cholesterol level and revealed that lovastatin has no obvious effect on promoting the excretion of cholesterol (Fig. 3e, f).

Finally, we asked whether the bile cholesterol could be excreted into the feces. Studies on cholesterol generally make it clear that cholesterol is primarily transformed into coprostanol by the intestinal microbiota [29–31]. Coprostanol comprised the largest amount of the feces and was produced from cholesterol principally by direct saturation of the 5, 6-double bond of cholesterol [32]. To clarify this point, we assessed the levels of cholesterol and coprostanol in feces. Compared with the ND hamsters, the level of cholesterol in the HFD hamsters’ feces at day 0 was significantly increased (*P* < 0.001), which may be due to a certain proportion of cholesterol in the high-fat diet. From day 0 to day 3, increased cholesterol and decreased coprostanol were observed in the feces of the HFD hamsters (Fig. 3g). From day 3 to day 10, decreased cholesterol and significantly increased coprostanol were observed (*P* < 0.001), which indicated that the bile cholesterol was ultimately excreted into the feces, primarily in the form of coprostanol.

### TBA in liver, bile and feces

**Fig. 4:**
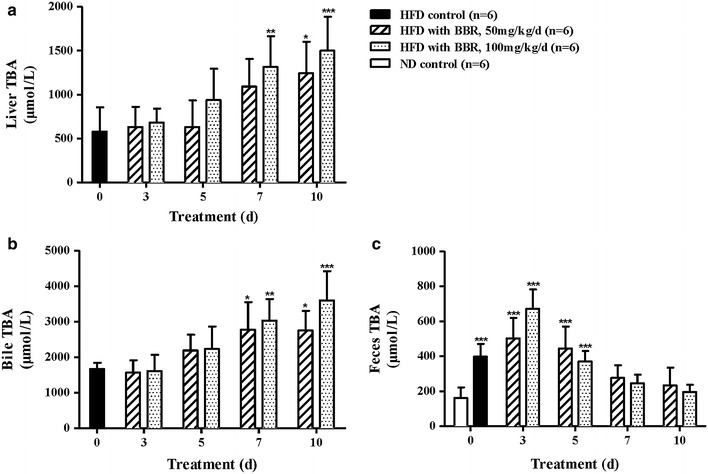
BBR increased TBA in both the livers and the bile of the hyperlipidemic hamsters. (**a**) BBR caused a significant increase in the liver TBA levels in the HFD hamsters. (**b**) The levels of bile TBA were markedly increased by BBR treatment. (**c**) In the feces, the levels of TBA were significantly decreased from day 3 to day 10. The data are shown as the mean ± SD. *P < 0.05, **P < 0.01 and ***P < 0.001 vs the HFD controls (**a**, **b**) or ND controls (**c**).

TBA is synthesized and catabolized from cholesterol by the liver. The levels of TBA in the liver, bile and feces were determined at the end of the study to demonstrate that BBR has evident effects on another aspect of cholesterol. As a result, BBR caused a significant increase in liver TBA in hyperlipidemic animals compared with the HFD control group (Fig. 4a), and the level of bile TBA was markedly increased (Fig. 4b). The liver is the main organ of cholesterol synthesis and clearance. When a large amount of cholesterol accumulated in the liver, BBR promoted the transformation of cholesterol to bile acids to a certain extent, thus resulting in more rapid excretion of cholesterol. However, in the feces, the level of TBA was increased in the BBR-treated HFD animals from day 0 to day 3 and significantly decreased from day 3 to day 10 (*P* < 0.001, Fig. 4c). After TBA is secreted by the liver cells into the bile, it enters the intestine with the bile flow and is then hydrolyzed to other forms of bile acid by the action of the intestinal microbiota. Finally, 97 % of the bile acid is returned to the liver after intestinal reabsorption. The whole process of the bile acid cycle is extremely complicated, and further study is necessary.

In general, this is the first study to determine the cholesterol in bile in BBR-treated hyperlipidemic animals. A high-fat diet was used to induce hyperlipidemia in hamsters to study the effects of BBR on cholesterol excretion. Using this hamster model, we demonstrated that BBR significantly promoted cholesterol excretion from the liver into the bile and finally excreted it into the feces, primarily in the form of coprostanol, which led to large decreases in the serum TC, TG and LDL-C levels. A recent study showed that BBR promoted the excretion of neutral and acidic sterols [33]. In another study, BBR lowered the blood cholesterol levels by inhibiting the intestinal absorption, with additional support provided by strong correlations between the cholesterol absorption rates and the plasma total or non-HDL cholesterol levels [20]. These are all different mechanisms that are distinct from the reported enhancement of the previously reported LDLR-mediated liver LDL-C clearance mechanism [1]. The most widely used drugs that stabilize and elevate LDLR are the PCSK 9 inhibitors, and BBR decreases the PCSK 9 mRNA and protein levels in a time- and dose-dependent manner. This was not due to increased degradation of the PCSK 9 mRNA but was most likely due to the decreased transcription of the PCSK 9 gene [34]. Our results did help prove that BBR is a multi-target lipid-lowering drug. We also compared BBR with lovastatin in several aspects, including the effects of lowering the serum lipid levels, inducing liver damage and promoting the excretion of cholesterol. As a result, our data show that BBR has an effective role in lowering the serum TC, TG and LDL-C levels, which may be superior to lovastatin in lowering the serum TC level. Moreover, 30 mg/kg/day lovastatin resulted in a certain degree of liver damage, whereas neither dose of BBR damaged the liver. Nevertheless, due to the poor absorption of BBR in vivo, we chose the effective doses of BBR as 50 and 100 mg/kg/day based on previous reports [1, 13]. Increasing the dose of lovastatin to be equal to BBR, with a dose of 50 mg/kg/day or even 100 mg/kg/day, will exceed the safe dose range of lovastatin. Therefore, the doses of BBR and lovastatin used here were not consistent [1, 28]. In addition, this may be the first study to elucidate that BBR can promote cholesterol excretion into the bile. Therefore, it will be very helpful to explore the cholesterol-lowering mechanism of BBR in the future.

## Conclusions

We conclude that BBR significantly promoted the excretion of cholesterol from the liver to the bile in hyperlipidemic hamsters, which led to large decreases in the serum TC, TG and LDL-C levels. There were no obvious side effects due to the serum transaminases after the BBR treatment, although high doses of lovastatin could influence the liver function to a certain extent. BBR is suggested to be useful as a monotherapy or in combination with statins.

## Abbreviations

BBR: berberine
ERK: extracellular-signal regulated kinase
LDLR: low-density lipoprotein receptor
HFD: high-fat diet
TC: total cholesterol
TG: triglyceride
LDL-C: low-density lipoprotein cholesterol
TBA: total bile acid
ELISA: enzyme-linked immunosorbent assay
OTC: over-the-counter
GC–MS: gas chromatography–mass spectrometry
mRNA: messenger ribonucleic acid
HMG-CoA: 3-hydroxy-3-methylglutaryl-coenzyme A
AMPK: AMP-activated protein kinase
PPAR: peroxisome proliferator-activated receptor
FFA: free fatty acid
CPK: creatine phosphokinase
ALT: alanine aminotransferase
AST: aspartate aminotransferase
GGT: glutamyltransferase
ND: normal diet
ANOVA: analysis of variance
S.D.: standard deviation
